# DLL4/Notch3/WNT5B axis mediates bidirectional prometastatic crosstalk between melanoma and lymphatic endothelial cells

**DOI:** 10.1172/jci.insight.171821

**Published:** 2024-01-09

**Authors:** Sanni Alve, Silvia Gramolelli, Joonas Jukonen, Susanna Juteau, Anne Pink, Atte A. Manninen, Satu Hänninen, Elisa Monto, Madeleine H. Lackman, Olli Carpén, Pipsa Saharinen, Sinem Karaman, Kari Vaahtomeri, Päivi M. Ojala

**Affiliations:** 1 Translational Cancer Medicine Research Program, Research Programs Unit, Faculty of Medicine, University of Helsinki, Helsinki, Finland.; 2 Faculty of Science and Engineering, Cell Biology, Åbo Akademi University, Turku, Finland.; 3 Turku Bioscience Centre, University of Turku and Åbo Akademi University, Turku, Finland.; 4 Department of Pathology, Helsinki University Hospital (HUS), University of Helsinki, Helsinki, Finland.; 5 Department of Plastic Surgery, Park Hospital, Helsinki University Hospital (HUS), and; 6 Individualized Drug Therapy Research Program, Faculty of Medicine, University of Helsinki, Helsinki, Finland.; 7 Helsinki Biobank, and; 8 Department of Pathology and Research Program in Systems Oncology, University of Helsinki, HUS Diagnostic Center, Helsinki University Hospital, Finland.; 9 Wihuri Research Institute, Biomedicum, Helsinki, Finland.; 10 Department of Biochemistry and Developmental Biology, Faculty of Medicine, University of Helsinki, Helsinki, Finland.

**Keywords:** Cell Biology, Vascular Biology, Cancer, Lymph, Melanoma

## Abstract

Despite strong indications that interactions between melanoma and lymphatic vessels actively promote melanoma progression, the molecular mechanisms are not yet completely understood. To characterize molecular factors of this crosstalk, we established human primary lymphatic endothelial cell (LEC) cocultures with human melanoma cell lines. Here, we show that coculture with melanoma cells induced transcriptomic changes in LECs and led to multiple changes in their function. WNT5B, a paracrine signaling molecule upregulated in melanoma cells upon LEC interaction, was found to contribute to the functional changes in LECs. Moreover, *WNT5B* transcription was regulated by Notch3 in melanoma cells following the coculture with LECs, and Notch3 and WNT5B were coexpressed in melanoma patient primary tumor and metastasis samples. Moreover, melanoma cells derived from LEC coculture escaped efficiently from the primary site to the proximal tumor-draining lymph nodes, which was impaired upon WNT5B depletion. This supported the role of WNT5B in promoting the metastatic potential of melanoma cells through its effects on LECs. Finally, DLL4, a Notch ligand expressed in LECs, was identified as an upstream inducer of the Notch3/WNT5B axis in melanoma. This study elucidated WNT5B as a key molecular factor mediating bidirectional crosstalk between melanoma cells and lymphatic endothelium and promoting melanoma metastasis.

## Abstract 2

A bi-directional DLL4/Notch3/WNT5B-dependent signaling cascade underlies crosstalk between melanoma and lymphatic endothelial cells and promotes melanoma lymphatic metastasis.

## Introduction

Metastatic melanoma is the most lethal form of skin cancer and its incidence continues to increase especially in the Western population. The prognosis for patients with metastasized disease is poor, with a long-term survival rate of only 10% (1). It is estimated that 80% of the melanoma metastases spread from the primary tumor to distant sites through the lymphatic vasculature. The importance of lymphatic vasculature for melanoma metastasis is further supported by observations that the peritumoral lymphatic vessel infiltration correlates with higher metastatic rate and thereby increased mortality in melanoma (2).

Tumors actively shape and modify the surrounding lymphatic system. Using 3D imaging of a mouse pancreatic cancer model, it was shown that the developing tumor caused extensive changes in the architecture of tumor-associated lymphatic vasculature, including remodeling of the existing lymphatic vasculature, invagination of the endothelium, and vasodilation (3). For melanoma, it has been demonstrated that cells can release lymphangiogenic factors to locally control lymphatic vasculature (4). Tumors not only cause changes in the local lymphatic vasculature but also in the tumor-draining lymph nodes; these lymph nodes appear larger due to proliferation of the resident macrophages and stromal lymphatic endothelial cells (LECs) (5). Furthermore, melanoma-secreted extracellular vesicles educate the tumor-draining lymph nodes to enhance lymphatic metastasis in many ways, for instance through neural growth factor receptor–dependent signaling (6); by shuttling tumor antigens to lymph node LECs for cross-presentation on MHC-I, resulting in apoptosis induction in antigen-specific CD8^+^ T cells (7); or by compromising the maturation process of the lymph node–residing dendritic cells (8).

Not only does melanoma actively remodel lymphatic vessels to promote cancer progression, but there is also increasing evidence that lymphatic vasculature can in turn directly modify the properties of melanoma cells, which suggests that active, bidirectional crosstalk occurs between the cancer cells and the lymphatic vasculature. For example, tumor-associated LECs can actively attract the cancer cells, leading to enhanced cancer cell migration toward the lymph vessels (9, 10). In addition, we have previously shown that direct contact of melanoma cells with LECs strongly augments melanoma invasion and metastasis through induction of Notch3 in the melanoma cells that is dependent on matrix metalloproteinase 14 (MMP14, also known as MT1-MMP) (11).

While the pivotal role of the lymphatic vasculature in promoting cancer dissemination is well conceded, the initial steps of lymphogenic cancer metastasis, and in particular the nature and molecular signaling involved in tumor cell communication with the surrounding lymphatic vasculature, are not yet completely understood. To better understand this reciprocal metastasis-promoting crosstalk, we implemented coculture systems of melanoma cells and LECs to discover molecular determinants for the communication between these 2 cell types. We found that melanoma cells induced metastasis-promoting molecular and functional changes in LECs mediated by the DLL4/Notch3/WNT5B signaling axis.

## Results

### Melanoma induces functional and transcriptional changes in LECs.

**Figure 1:**
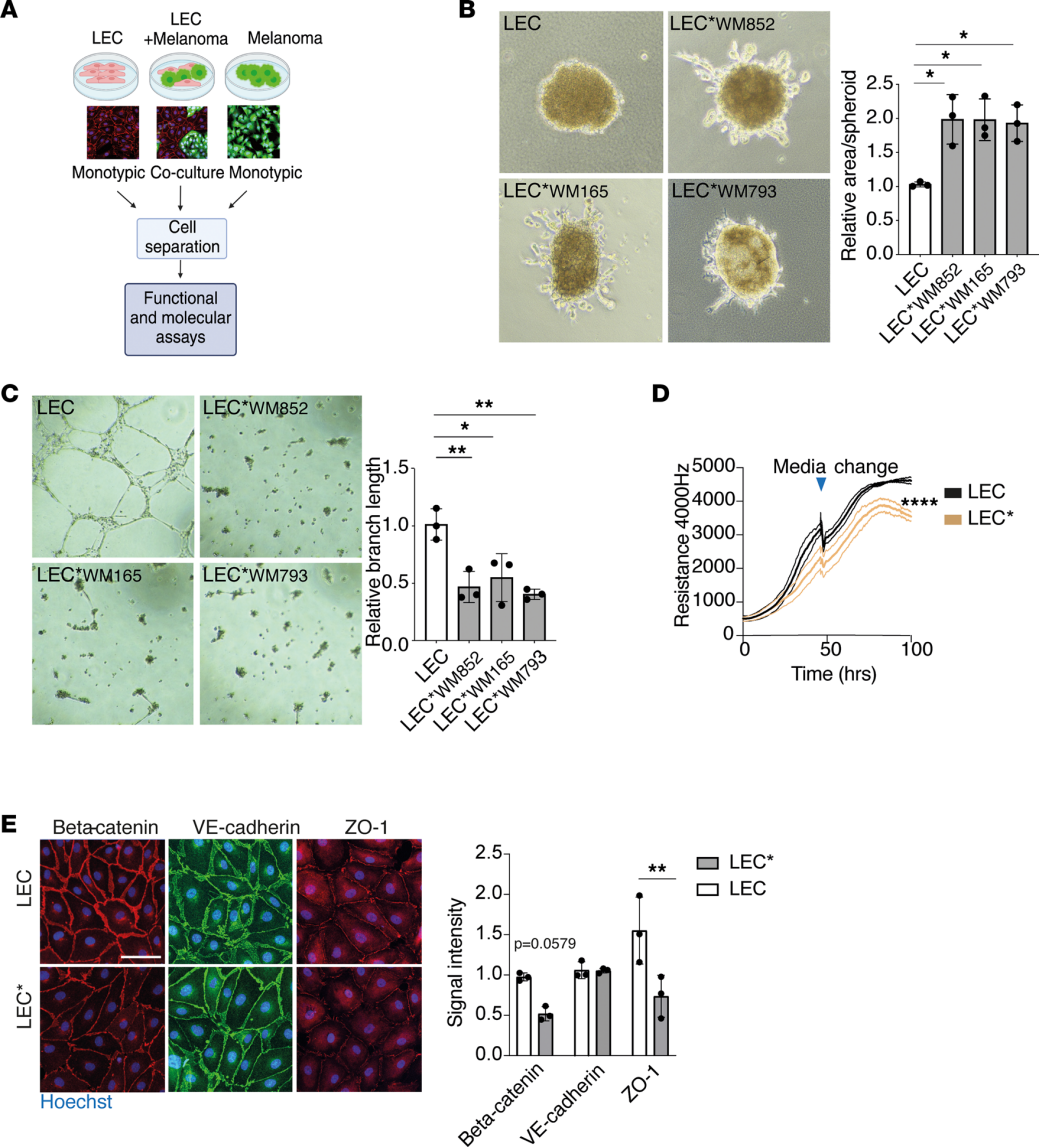
Melanoma cells induce functional changes in LECs. (**A**) Schematic of the workflow for the monotypic and coculture cell models. Representative immunofluorescence images are shown, with green representing the GFP-expressing melanoma cells and red the LECs labeled with anti-CD31. Nuclei were counterstained with Hoechst 33342 (blue). Figure generated by BioRender.com. (**B**) Spheroid-sprouting assay of LECs cultured as a monotypic control culture (LEC) or as a melanoma cell coculture (LEC* followed by name of the utilized melanoma cell line) for 2 days before cell separation by FACS. Representative images of spheroids after 4 days in fibrin are shown. Quantification of sprouting area from 3 independent experiments of at least 3 spheroids per condition is shown in the right panel. (**C**) Tube formation assay of LECs and LECs* cultured and sorted as in **A** and seeded on Cultrex for 16 hours. Representative images from 3 independent experiments are shown on the left panel and quantification of branch length on the right panel. (**D**) Monotypic control LECs and LECs* originating from a coculture with WM852 melanoma cell line were analyzed by an electrical cell impedance assay after 2 days of culture. A representative assay of 2 independent experiments is shown. Thicker lines indicate mean values and thinner lines ±SD. (**E**) Immunofluorescence images of the indicated proteins of monotypic LECs or LECs* originating from a coculture with the WM852 melanoma cell line. Nuclei were counterstained with Hoechst 33342. Representative images from 3 independent experiments are shown. Scale bar: 50 μm. Data are presented as mean ± SD. *P < 0.05, **P < 0.01, ****P < 0.0001 by 1-way ANOVA followed by Tukey’s multiple-comparison test (**B**, **C**, and **E**) or AUC analysis followed by unpaired, 2-tailed *t* test (**D**).

To characterize possible changes in LECs upon their coculture with melanoma cells, we used a combination of 2D and 3D functional assays to assess the LEC properties. For each of these experiments, LECs were cultured either in monotypic control cultures or cocultured with a panel of GFP-expressing melanoma cell lines: both metastatic (WM852 and WM165) and nonmetastatic (WM793). After 2 days in coculture, cells were sorted and the LECs used for subsequent assays (indicated as LEC*; Figure 1A).

First, we utilized a spheroid-sprouting assay where preformed LEC spheroids were embedded into a cross-linked 3D fibrin matrix. Fibrin was chosen since it is frequently deposited within the melanoma tumor microenvironment and perivascular niche in vivo (12). During the 4-day incubation in 3D matrix, the monotypic control LEC spheroids remained as round spheres, while an outgrowth of sprouts was observed in the LEC* spheroids derived from melanoma cell cocultures with all the cell lines tested (Figure 1B), indicating a clear phenotypic change in the LECs after the melanoma cell coculture.

To determine the capacity of LECs to form capillary-like structures, we exploited a classical tube formation assay in which LECs are cultured overnight on a reconstituted basement membrane extracellular matrix support (Cultrex). Monotypic LECs spontaneously formed tube-like structures suggestive of a clear vasculogenic cell behavior, whereas the LECs* originating from melanoma cell cocultures largely failed to form continuous tubular networks (Figure 1C).

LEC monolayer permeability as an indicator of its barrier function was addressed using noninvasive electrical cell impedance monitoring. LECs* from coculture with WM852 and WM165 melanoma cells showed an impaired ability to form insulating barriers compared with the control LEC layers (Figure 1D and Supplemental Figure 1A; supplemental material available online with this article; https://doi.org/10.1172/jci.insight.171821DS1). This suggests that cell-cell contacts are weakened upon coculture with melanoma cells. Therefore, we next analyzed expression of well-known proteins at the cell-cell contact sites in the monotypic control LECs and LECs*. In line with this, after melanoma cell coculture, significantly less β-catenin and ZO-1 signal was detected in LECs* when compared with the parental, monotypic control LECs (Figure 1E). Although we did not see significant reduction in VE-cadherin intensity at the cell junctions, its staining pattern in LECs* was less serrated and reticular, possibly indicating weaker cell-cell junctions (13, 14). These results suggest that melanoma cell interaction with LECs may induce changes in the LEC junction maturation processes.

Lastly, we addressed the effect of coculture on LEC proliferation (Supplemental Figure 1B). After cell sorting, monotypic control LECs and LECs* cocultured with the WM852 melanoma cell line were subjected to EdU-based click-it chemistry to identify proliferating cells. No significant changes in the ratio of proliferating cells were seen between the monotypic control and LECs*.

Taken together, these results show that clear phenotypic and functional changes were observed in LECs* after the coculture with melanoma cells. The changes might at least partly be due to changes in the cell-cell contacts, as suggested by the weaker ability of the LECs* to form insulating barriers and the altered levels and distribution of the analyzed cell junctional proteins.

**Figure 2:**
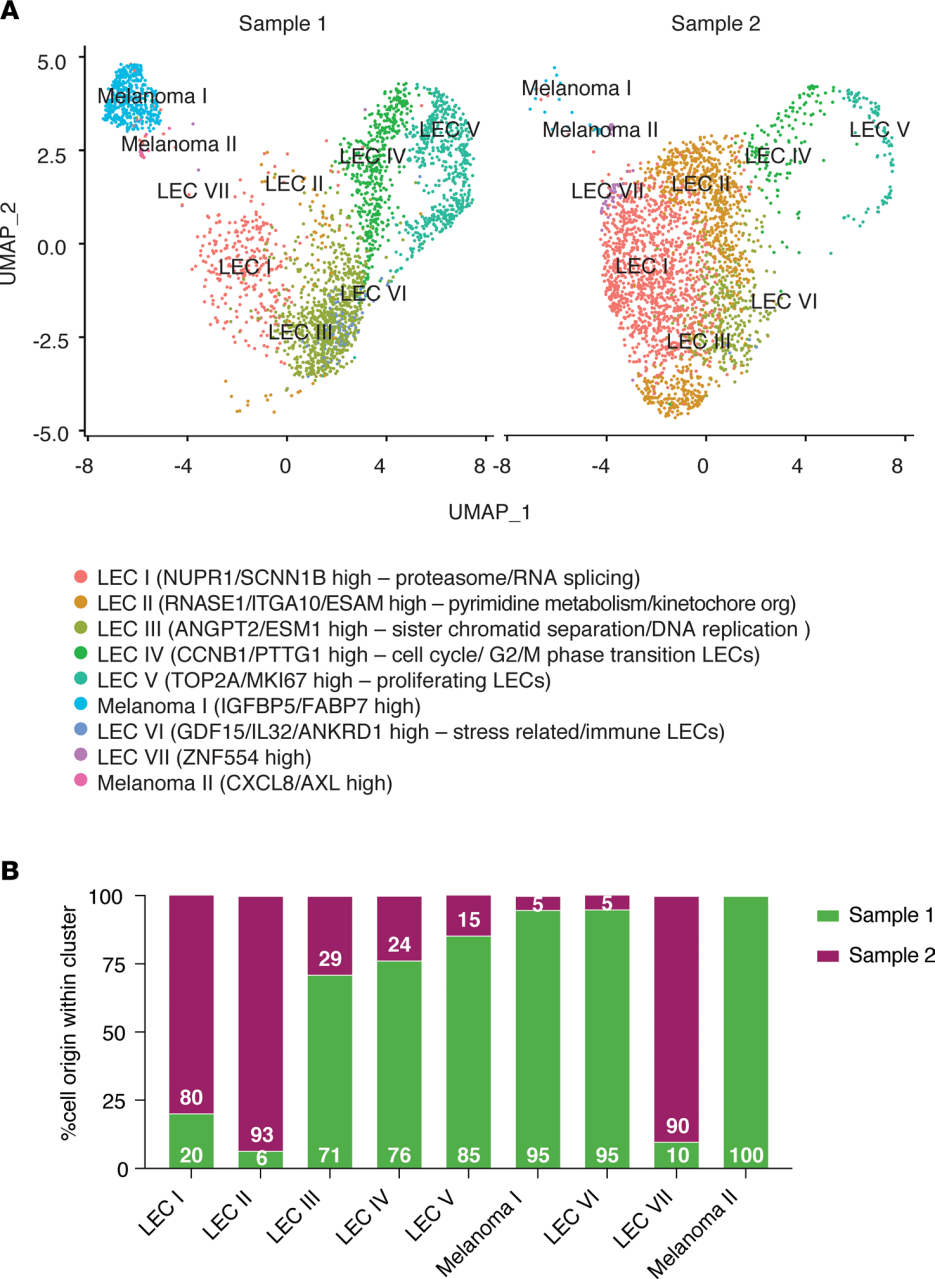
Melanoma cells induce gene expression changes in LECs. (**A**) UMAP clustering plots with the corresponding annotations of sample 1, consisting of a mixture of monotypic control LECs and monotypic WM852 melanoma cells, and sample 2 consisting of LECs* cocultured for 2 days with WM852 melanoma cells and separated by FACS for analysis. The cell clusters were annotated based on upregulated marker genes and pathways that were found to be altered by differential gene expression analysis. (**B**) Distribution of the cells in samples 1 and 2 within the clusters.

To uncover molecular changes in LECs induced by the melanoma cell coculture, we performed single-cell RNA sequencing (scRNA-seq) to compare the gene expression profiles of monotypic LECs (control LECs) to LECs* cocultured with the metastatic melanoma cell line WM852. To that end, 2 different samples were prepared and analyzed by scRNA-seq. First, LECs from a monotypic culture were mixed with WM852 melanoma cells (1:10 melanoma/LEC ratio) from a monotypic culture (sample 1, Figure 2A) in order to identify the possible residual melanoma cells originating from the coculture samples after separation of the 2 cell types. For the LEC* sample, LECs were cocultured for 2 days with WM852 melanoma cells, after which the 2 cell types were separated and the LECs* subjected to further analysis (sample 2, Figure 2A). Upon analysis and clustering in UMAP plots, 9 different cell clusters could be identified (Figure 2A). Feature heatmaps of melanoma (*SOX10*) and LEC (*PROX1*) markers showed that melanoma cells localized to 2 clusters (Melanoma I and II), and the remaining clusters (LEC I–VII) consisted of LECs and LECs* (Figure 2A and Supplemental Figure 2A). When the distribution of LECs within the clusters was analyzed, we found that in some clusters either the LECs* (sample 2) or control LECs (sample 1) were heavily enriched (Figure 2B). For example, cluster LEC II mainly consisted of the LECs* (sample 2), whereas cluster LEC III predominantly represented the control LECs (sample 1). The markers for each cluster are shown in Supplemental Table 1. Differential gene expression analysis of the scRNA-seq data (Supplemental Table 2) suggests that in both LEC I and LEC II, the renin-angiotensin GO term was enriched upon coculture (specifically genes *CTSA* and *PRCP*). On the other hand, in both clusters, pyrimidine metabolism is the most enriched GO term in the downregulated genes (*NT5E*, *TK1*, *DTYMK*, *TYMS*, and *NME1*), suggesting a suppression of cell proliferation possibly related to changes in the differentiation state of LECs following the coculture. By real-time quantitative PCR (qRT-PCR), we further validated selected genes that were found highly upregulated in the LEC* sample and involved in vascular development (*EDN1* and *ENG*) or inflammatory response (*CCL2* and *IL32*) pathways (Supplemental Figure 2B).

These results indicate that melanoma coculture induces phenotypic changes in LECs, including alteration of cell sprouting in 3D, angiogenic potential, and barrier functions and marked changes in gene expression levels of the pathways likely regulating these processes.

### Melanoma cell–derived WNT5B contributes to the functional changes in LECs.

**Figure 3:**
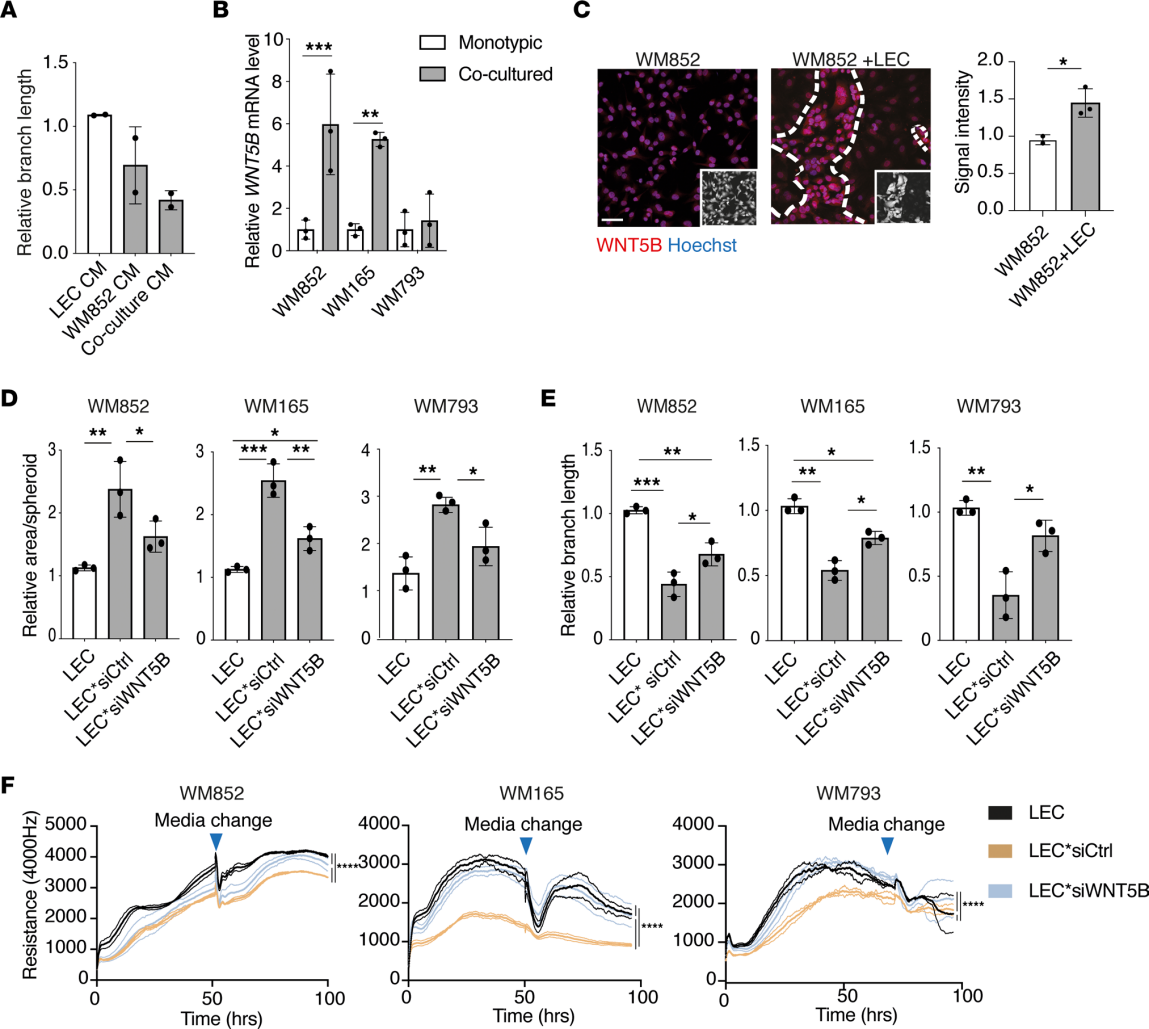
Melanoma cell–derived WNT5B contributes to the functional changes in LECs. (**A**) Quantification of the relative branch length of a tube formation assay with LECs cultured in conditioned media (CM) from monotypic LECs, WM852 cells, or LEC+WM852 coculture for 24 hours and analyzed by a 16-hour tube formation assay. Experiment was performed 2 independent times. (**B**) qRT-PCR of *WNT5B* mRNA levels in the indicated monotypic or LEC-cocultured melanoma cell lines from 3 independent experiments. (**C**) Immunofluorescence images of monotypic WM852 and WM852+LEC cultures labeled with an antibody against WNT5B. Small inserts identify the GFP-expressing melanoma cells. Nuclei were counterstained with Hoechst 33342. Representative images from 3 independent experiments are shown in the left panel, and quantification of WNT5B relative signal intensity from at least 100 cells/experiment/condition is shown in the right panel. Scale bar: 50 μm. (**D**) Quantification of the spheroid-sprouting assay of monotypic LECs and LECs cocultured with the indicated melanoma cells (LEC*). Prior to the coculture, melanoma cells were pretreated with control (siCtrl) or *WNT5B*-targeting siRNAs (si*WNT5B*) for 24 hours. Graph shows the mean of 3 independent experiments, each with at least 4 spheroids/condition quantified. WM852 and WM165 melanoma cell–LEC cocultures were performed at the same time and therefore the same LEC control spheroids were used for analysis. (**E**) Quantification of the tube formation assay with LECs cultured and treated as in **D** (*n* = 3). WM165 and WM793 melanoma cell–LEC cocultures were performed at the same time and therefore the same LEC control samples were used for analysis. (**F**) The electrical cell impedance assay with LECs cultured and treated as in **D**. Data are presented as mean ± SD for each sample. Representative experiment of 2 experiments is shown. *P < 0.05; **P < 0.01; ***P < 0.001; ****P < 0.0001 by 1-way ANOVA followed by Tukey’s multiple-comparison test (**A**, **B**, and **D**), unpaired, 2-tailed *t* test (**B** and **C**), or AUC analysis followed by 1-way ANOVA with Tukey’s multiple-comparison test (**F**).

To elucidate how the melanoma cells induce the observed changes in LECs*, we first addressed whether they require direct cell-cell contact between the 2 cell types or are mediated by a paracrine factor(s). To that end, we carried out tube formation assays in which the control LECs were cultured in conditioned media (CM) derived from either the monotypic cultures of LECs or WM852 or from the WM852-LEC coculture. The ability of LECs to form tubular structures was already disrupted when they were cultured in media originating from the monotypic melanoma cell culture. The tube formation capacity was disturbed to a greater extent with CM from the WM852-LEC coculture (Figure 3A). This suggested that a secreted factor(s) from the melanoma cells could be responsible for causing this functional change in LECs.

To identify the factor(s), we searched for possible candidates from our previously published RNA-seq data, where gene expression profiles of melanoma cells before and after the LEC coculture had been determined (11). In this data set, *WNT5B*, a gene encoding a secreted signaling molecule, was found to be highly upregulated in the LEC-cocultured WM852 melanoma cells. The role of WNT5B in cancers has not been extensively studied, but there are indications of WNT5B having tumor-promoting roles. For instance, WNT5B promotes proliferation and invasion of certain breast cancer and oral squamous cell carcinoma cell lines (15–17), in genomic analyses it associates with the most aggressive pancreatic cancer subtype (18), and pancreatic cancer cells that have undergone mesenchymal transition have been shown to promote the metastatic potential of the neighboring epithelial cells (19).

We next confirmed that *WNT5B* mRNA was upregulated upon LEC coculture in the metastatic melanoma cell lines WM852 and WM165. We also analyzed *WNT5B* induction following LEC coculture with a nonmetastatic melanoma cell line, WM793 (Figure 3B), but did not see any increase in *WNT5B* mRNA. Further analysis showed that this cell line expressed *WNT5B* at an approximately 10-fold higher level when compared with WM852 and WM165 cell lines already before the LEC coculture (Supplemental Figure 3A), suggesting different, intrinsic regulation of WNT5B expression in this cell line. The upregulation of WNT5B protein in the cocultured WM852 and WM165 cells was also confirmed by immunofluorescence (Figure 3C and Supplemental Figure 3B).

To investigate whether the melanoma cell–derived WNT5B could be responsible for the changes observed in the LEC* phenotype and function, melanoma cells were pretreated with siRNA targeting the *WNT5B* gene or control siRNA prior to the coculture with LECs. Coculture with the melanoma cells depleted of *WNT5B* (Supplemental Figure 3C) significantly reduced the sprouting of LEC spheroids (Figure 3D). Depletion of *WNT5B* in the melanoma cells could partially restore the tube formation ability of LECs* (Figure 3E) and the barrier function of the LEC* layer (Figure 3F). Accordingly, treatment with recombinant WNT5B protein was sufficient to reduce the barrier function of the control LEC layer (Supplemental Figure 3D) and decrease β-catenin and ZO-1 expression on the cell membrane (Supplemental Figure 3E). Together, these functional assays demonstrate that WNT5B expressed and secreted by the melanoma cells contributes to the functional changes in LECs*.

WNT ligands signal through binding to the Frizzled receptors and additional co-receptors on the target cell membrane (20). To investigate which receptor on the LEC surface would be responsible for transmitting the WNT5B signal, we first explored our scRNA-seq data for expression of *FZD* mRNAs in the different LEC clusters in Figure 2. As shown in Supplemental Figure 4A, *FZD4* and *FZD6* expression was found in most of the LEC clusters. There were no significant differences in the abundance of the transcripts between the control and cocultured samples. To validate these findings, we next characterized the mRNA expression of the 10 known human Frizzled receptors in the parental, control LECs and cocultured LECs* by qRT-PCR (Supplemental Figure 4B). Out of these receptors, 5 were expressed at detectable levels in LECs. *FZD6* and *FZD8* were the most abundantly expressed receptors in LECs and *FZD4* to a lesser extent. However, except for a 2-fold increase in *FZD1,* we did not observe significant differences in any of the Frizzled receptor mRNAs between the control LECs and LECs*. As previously shown by molecular docking experiments, WNT5B has the highest binding affinity for FZD8 (21). We therefore chose to focus on FZD4, FZD6, and FZD8 in the further experiments and treated the LECs with either control siRNA or siRNAs targeting the *FZD4*, *FZD6*, or *FZD8* gene for 24 hours before the start of the 48-hour coculture with WM852 melanoma cells, followed by sorting and functional assays. We were not able to generate intact spheroids following si*FZD4* treatment of LECs, most probably due to lower levels of β-catenin and VE-cadherin in the si*FZD4*-treated samples (Supplemental Figure 4C). Moreover, no differences between the LECs* and control LECs were observed in the spheroid-sprouting assay upon si*FZD6* or si*FZD8* treatment or tube formation assay upon si*FZD6*, si*FZD8*, or si*FZD4* treatment (Supplemental Figure 4, D and E). This suggests that there are other receptors or contributions by multiple receptors mediating the downstream effects of WNT5B in LECs.

### WNT5B facilitates melanoma cell escape from the primary injection site.

**Figure 4:**
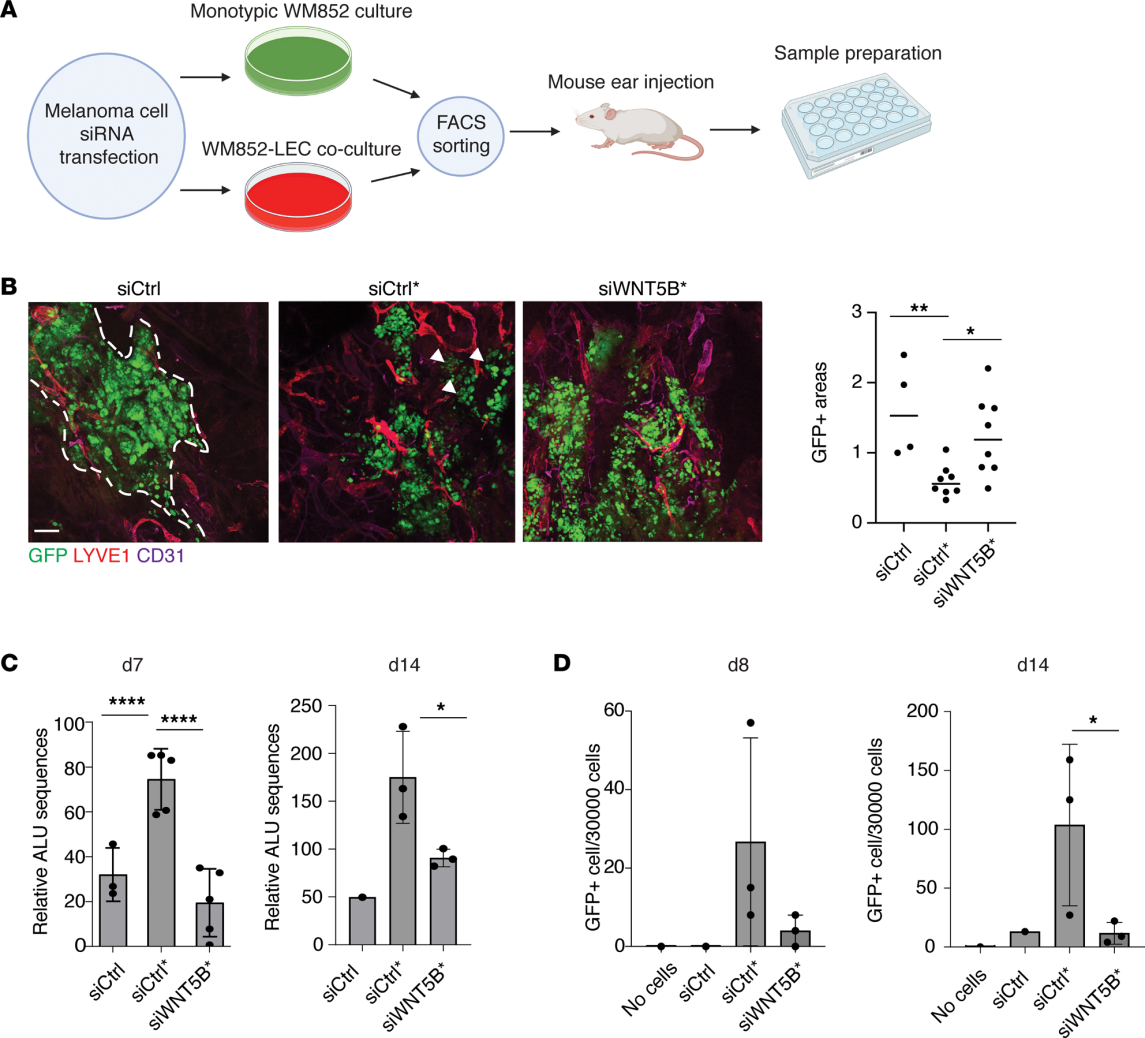
WNT5B facilitates melanoma cell escape into draining lymph nodes. (**A**) Schematic of the workflow. WM852 melanoma cells were treated with siRNAs for 24 hours and cultured as monotypic cultures (siCtrl) or with LEC (siCtrl*, siWNT5B*). After 2 days, the 2 cell types were separated and melanoma cells were injected intradermally into mouse ear pinna. After 1 week, mice were sacrificed and the ears, lungs, liver, and superficial and inguinal lymph nodes were harvested and processed for analyses. Schematics generated with BioRender.com. (**B**) Representative images of the GFP-expressing WM852 melanoma cells (siCtrl, siCtrl*, siWNT5B*) in mouse ear pinna epidermis. Dashed line indicates the boundaries of injected melanoma cells and arrowheads show the diffuse growth phenotype of the siCtrl* melanoma cells. The relative size of areas occupied by GFP^+^ melanoma cells was quantified from each mouse ear. Relative size for the GFP^+^ area of each mouse ear is shown (siCtrl, *n* = 4; siCtrl*, *n* = 8; siWNT5B*, *n* = 8; where *n* refers to number of ears quantified). Scale bar: 200 μm. (**C**) qPCR for the relative human Alu sequences from the mouse superficial cervical lymph nodes. Mouse genomic actin was used as a control. Single values for each mouse are shown. Day 7 (d7): siCtrl, *n* = 3; siCtrl*, *n* = 5; siWNT5B*, *n* = 5. d14: siCtrl, *n* = 1, siCtrl*, *n* = 3, siWNT5B*, *n* = 3. (**D**) Mouse ear xenografts were generated from WM852 cells as described in **A**. Superficial cervical lymph nodes were harvested after 8 and 14 days and analyzed by FACS for the presence of GFP^+^ tumor cells. Lymph nodes from untreated mice (no cells) were used as controls. Single values for each mouse are shown (siCtrl, *n* = 1; siCtrl*, *n* = 3; siWNT5B*, *n* = 3). Data are presented as mean ± SD. *P < 0.05, **P < 0.01, ****P < 0.0001 by 1-way ANOVA followed by Tukey’s multiple-comparison test (**B** and **C**, d7 samples) or 1-tailed *t* test (panel **C** d14 samples and **D**).

To further characterize the role of WNT5B in melanoma progression, we chose to use an in vivo tumor model where melanoma cells are injected intradermally into the mouse ear pinna, which is rich in lymphatic capillary networks and feasible to image by confocal microscopy (22). To that end, we first treated WM852 melanoma cells with control siRNA or siRNA targeting *WNT5B* prior to the 48-hour coculture with LECs and subsequent cell separation (Figure 4A). Cells (5 × 10^5^) from each condition were implanted in Matrigel and allowed to grow for 1 or 2 weeks, after which the mice were sacrificed, and the ears processed for whole-mount immunofluorescence and imaging. After 7 days, significantly fewer cells were seen in the ear samples injected with the LEC-cocultured melanoma cells (siCtrl*) compared with the injection sites of monotypic control melanoma cells (siCtrl) (Figure 4B). Moreover, the siCtrl* melanoma cells displayed a diffuse growth phenotype (see arrowheads in Figure 4B) when compared with the siCtrl-treated melanoma cells (indicated with dashed line in Figure 4B), suggesting a more invasive character of these LEC-cocultured cells (11). Since we had not seen significant differences in the proliferation rates between the siCtrl* and siCtrl cells in vitro (Supplemental Figure 5A), this suggested that the siCtrl* melanoma cells might have escaped from the primary injection site via the lymphatic vasculature more efficiently than the siCtrl cells.

When cells pretreated with siRNA targeting *WNT5B* (siWNT5B*) prior to the coculture were injected, they were more extensively retained at the initial injection site when compared with the siCtrl* cells (Figure 4B). Interestingly, the implanted siWNT5B* cells showed a similar, diffuse growth phenotype comparable to that of siCtrl* cells, indicating that the melanoma-derived WNT5B was primarily affecting the passage through lymphatic vasculature rather than the growth phenotype of melanoma cells at the primary tumor site.

We next hypothesized that the siCtrl* cells would first locally invade the lymphatic capillaries in the ear, and further drain into the cervical sentinel lymph nodes in the mouse neck. To assess this, we harvested superficial cervical and inguinal lymph nodes and quantified the human Alu sequences as an indication of the presence of human melanoma cells in the lymph nodes (Figure 4C and Supplemental Figure 5B). As a negative control we used mouse inguinal lymph nodes (Supplemental Figure 5B), since we did not expect that the short duration of the experiment would be sufficient for melanoma cells to metastasize from the initial injection site to more distant lymph nodes. The relative Alu sequence amount was significantly higher in the superficial cervical lymph nodes collected from the mice implanted with siCtrl* melanoma cells when compared with the siCtrl ones, supporting more efficient escape of the siCtrl* cells from the primary injection site (Figure 4C). Importantly, on days 7 and 14 after injection, significantly less Alu sequence signal was found in the superficial cervical lymph nodes of the mice implanted with siWNT5B* cells when compared with mice implanted with siCtrl* cells (Figure 4C).

To provide further evidence of a true lymph node metastasis of the siCtrl* cells and its dependence on WNT5B, we analyzed the presence of GFP-expressing siCtrl, siCtrl*, and siWNT5B* WM852 cells in the superficial cervical and inguinal lymph nodes by FACS 8 and 14 days after melanoma cell injection. In accordance with the Alu sequence signal, the highest numbers of cells were detected in the lymph nodes on days 8 and 14 from mice injected with siCtrl* cells when compared with siCtrl- and siWNT5B*-treated cells, and after 14 days there were significantly more melanoma cells present in the siCtrl* samples compared with siWNT5B* ones (Figure 4D). We did not observe any GFP-expressing tumor cells in the inguinal lymph nodes (Supplemental Figure 5C).

These data demonstrate that WNT5B-mediated melanoma-LEC crosstalk plays an important role in promoting melanoma metastasis in vivo from the primary injection site to the sentinel lymph nodes, most probably through its effects on the LECs.

### Notch3 regulates WNT5B expression in melanoma cells.

**Figure 5:**
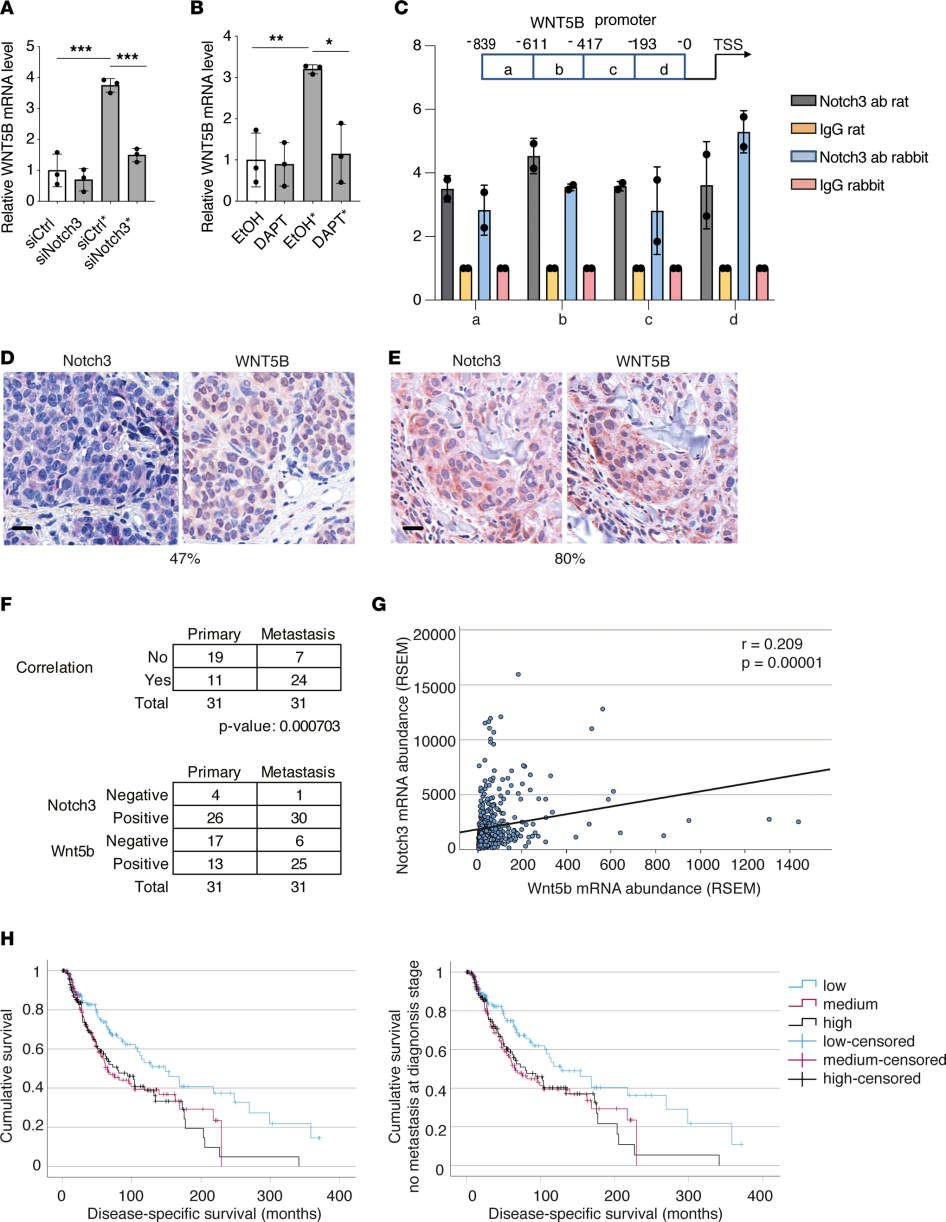
Notch3 regulates WNT5B expression in melanoma cells and its expression correlates with poor disease-specific survival. (**A**) WM852 cells were pretreated with the indicated siRNAs and subjected to monotypic or LEC cocultures (*). After cell sorting, *WNT5B* mRNA levels in melanoma cells were measured by qRT-PCR. Graph shows results from 3 independent experiments. (**B**) WM852 cells were cultured as monotypic cultures or in coculture with LECs (*) and treated with vehicle (EtOH) or DAPT. *WNT5B* mRNA level was measured in the sorted melanoma cells by qRT-PCR (*n* = 3). (**C**) Top: Schematic presentation of the *WNT5B* promoter area amplified by qPCR following ChIP. Numbers indicate the nucleotides upstream of the *WNT5B* transcription start site. Bottom: ChIP from WM852 cells expressing ectopic NICD3 for 24 hours. ChIP was performed with 2 different anti-Notch3 antibodies and respective control IgGs and DNA was amplified from the indicated promoter regions of the *WNT5B* gene (*n* = 2). Data in **A**–**C** are presented as mean ± SD. (**D** and **E**) Representative images of human primary melanoma tumor (**D**) and metastasis samples (**E**) labeled for the indicated proteins. Percentage below the images indicates the proportion of samples with Notch3 and WNT5B signal colocalization. Scale bars: 20 μm. (**F**) Statistical analysis of co-distribution in primary tumors and metastatic samples. (**G**) RSEM-quantitated mRNA abundance comparing *NOTCH3* and *WNT5B* shows a significant, positive correlation between the 2 gene transcripts. The *P* value was obtained using the 2-tailed *t* test for Pearson’s correlation coefficient, *r*. (**H**) Survival statistics were computed for the entire cohort of 442 melanoma patients (left panel), or for the cases with no distant metastases at the time of diagnosis (right panel). Patients were divided by their *NOTCH3* mRNA level expression into 3 groups based on RSEM-quantitated mRNA abundance percentiles: Low, first to 33rd percentile; Medium, 34th to 67th percentile; High, 68th to 100th percentile. Log-rank *P* values for the left panel: 0.004, right panel: 0.013. *P < 0.05; **P < 0.01; ***P < 0.001 by 1-way ANOVA followed by Tukey’s multiple-comparison test (**A** and **B**) or 2-tailed *t* test (**F**).

We have previously shown that Notch3 is highly upregulated in melanoma cells upon coculture with LECs and is important for melanoma invasion and metastasis (11). Interestingly, Notch3 binding to the *WNT5B* promoter area has been previously reported, but not validated, in ovarian cancer cells (23). Therefore, we tested whether Notch3 would function as the upstream regulator of WNT5B expression in the cocultured melanoma cells. To that end, we set up cocultures of LECs with WM852 and WM165 melanoma cells pretreated with control siRNAs (siCtrl*) or siRNAs targeting *NOTCH3* 24 hours prior to the start of the 48-hour coculture. The siCtrl-treated monotypic melanoma cells were used as a control. When compared with the siCtrl* cells, the *WNT5B* mRNA was significantly reduced in the siNOTCH3*-pretreated cell lines (Figure 5A and Supplemental Figure 6A), suggesting that Notch3 was activating *WNT5B* transcription. *NOTCH3* depletion in the monotypic melanoma cells, however, did not significantly affect the basal level of *WNT5B* (Figure 5A and Supplemental Figure 6A). To confirm the role of Notch3 as an inducer of *WNT5B* expression, the coculture assays were also carried out in the presence of the Notch pathway inhibitor DAPT or vehicle as a control (ethanol, EtOH).

When compared with vehicle-treated cocultures, the DAPT treatment significantly reduced the upregulation of *WNT5B* mRNA levels in WM852 and WM165 cells cocultured with LECs (Figure 5B and Supplemental Figure 6B), further supporting a key role for Notch3 in regulating WNT5B expression in the metastatic WM852 and WM165 melanoma cells. However, the nonmetastatic melanoma cell line WM793, with endogenously high *WNT5B* levels, did not show any significant change in the *WNT5B* mRNA levels upon si*NOTCH3* or DAPT treatment, suggesting regulatory pathways other than Notch3 for the sustained, high WNT5B expression in this cell line (Figure 5, A and B, and Supplemental Figure 6, A and B).

To confirm the role of Notch3 as a transcriptional activator of *WNT5B*, we next addressed the binding of Notch3 on the *WNT5B* promoter region by ChIP-PCR. To that end, WM852 melanoma cells were first transfected with a plasmid encoding an active Notch3 intracellular domain, NICD3, and subjected to ChIP on day 2 using 2 different anti-Notch3 antibodies. As shown in Figure 5C, Notch3 binds to the *WNT5B* gene promoter region, thus confirming its role as a transcriptional activator of WNT5B in melanoma cells. Together, these data demonstrate that Notch3 acts as an upstream positive regulator of WNT5B in the metastatic melanoma cell lines WM852 and WM165.

We next investigated whether Notch3 and WNT5B expression would correlate in melanoma patient samples (Supplemental Table 3). We chose to use a patient cohort, where, despite a confirmed negative sentinel lymph node biopsy at the time of diagnosis, the patients later presented with metastases. At the time of the diagnosis, 36 out of 55 patients had tumors less than 4 mm deep as measured by the Breslow thickness, thus representing patients in whom the sentinel lymph node involvement is routinely used as a diagnostic tool. This cohort was also atypical, since the patients’ age at the time of diagnosis did not correlate with survival time. Their disease progressed very aggressively, with an average survival of 3.75 years (Supplemental Figure 6C), in comparison with an average 5-year survival of 99.5% for a patient with localized melanoma (https://seer.cancer.gov/statfacts/html/melan.html). Fifty-five primary tumor samples and 44 metastasis samples from these 55 melanoma patients were stained with antibodies against WNT5B and Notch3. Forty-seven samples from the primary tumors and 35 from the metastases were successfully scored. Furthermore, we had a total of 31 patients from whom both the primary and metastasis sample pairs were successfully scored for both Notch3 and WNT5B expression. Co-distribution of expression of the 2 proteins was found in 35% of the primary tumor samples (Figure 5D) and in 77% of the samples from the first site of metastasis (Figure 5E). For assessing the statistical significance of the coexpression of Notch3 and WNT5B in the primary and metastasis stages, a paired-sample *t* test was performed and demonstrated that metastatic samples showed significantly more correlation for Notch3 and WNT5B coexpression compared with the primary tumors (Figure 5F), providing further support for the importance of the Notch3/WNT5B axis in melanoma metastasis. Furthermore, expression of Notch3 and WNT5B shows a significant, positive correlation at the mRNA level in a 442-patient melanoma data set obtained from The Cancer Genome Atlas (TCGA) (24) (Figure 5G). This further supports our findings from the functional assays that the Notch3/WNT5B axis has an important role in melanoma aggressiveness.

To look for further evidence for the importance of Notch3 and WNT5B in the aggressiveness of melanoma, we performed Kaplan-Meier survival association analyses for both genes using the 442 patient cohort from TCGA. In this analysis, Notch3 expression significantly correlated with poor survival in the entire cohort and in the patients who did not have distant metastases at the time of diagnosis (Figure 5H). This highlights the role of Notch3 in the capacity for initiating metastasis, even in a cohort with melanoma cases, which are less aggressive than the extremely aggressive cases in our cohort. WNT5B did not significantly correlate with patient survival, potentially due to Notch3 being the upstream regulator of WNT5B transcription and production in the melanoma cells.

### Notch ligand DLL4 is a potent inducer of Notch3 and WNT5B in melanoma.

**Figure 6:**
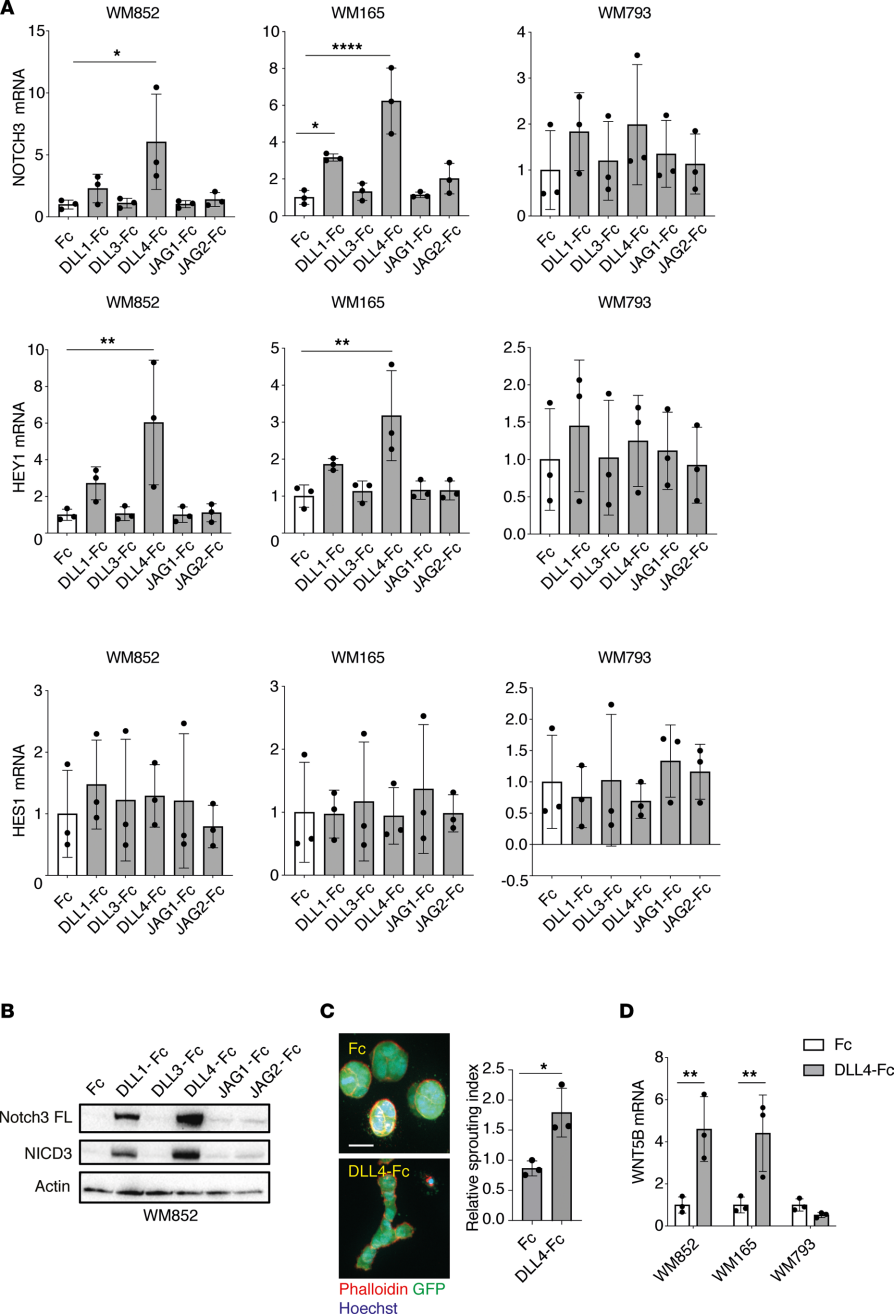
Notch ligand DLL4 is a potent inducer of Notch3 and WNT5B in melanoma. (**A**) Indicated melanoma cell lines cultured on dishes precoated with the indicated chimeric Fc fused with Notch ligands or Fc alone as a control for 2 days, and analyzed by RT-qPCR for *NOTCH3*, *HEY1*, and *HES1* (*n* = 3). (**B**) Immunoblotting of WM852 cells cultured as in **A** for the indicated targets. FL, full length. A representative blot of 3 independent experiments is shown. (**C**) A 3D fibrin droplet invasion assay of WM852 cells cultured on DLL4-Fc and Fc as in **A**. GFP-expressing melanoma cells were stained with Phalloidin 594 and nuclei were counterstained with Hoechst 33342. Maximum intensity of *z* projections of the confocal stacks are shown. Graph shows quantification of the relative invasive index from 3 independent experiments with at least 50 cell clusters quantified/condition. Scale bar: 20 μm. (**D**) qRT-PCR of *WNT5B* levels in melanoma cell lines cultured as in **C**. Experiment was performed 3 independent times. Data are presented as mean ± SD. *P < 0.05, **P < 0.01, ****P < 0.0001 by 1-way ANOVA followed by Dunnett’s multiple-comparison test (**A**) or 2-tailed *t* test (**C** and **D**).

We next investigated how Notch3 activation is induced in the melanoma cells upon interaction with LECs in coculture. Notch signaling is initiated when the Notch receptor binds to its ligand, a transmembrane protein on the cell membrane of a neighboring cell. This induces a pulling force on the Notch receptor, thus exposing the receptor’s 2 protease cleavage sites. The cleavage allows the release of the intracellular domain of the receptor (NICD) and its nuclear translocation where it binds to additional cofactors to initiate target gene transcription (25). Human cells express all 5 Notch ligands, DLL1, DLL3, DLL4, JAG1, and JAG2 (26). We therefore first addressed which of these ligands are expressed in LECs and LECs*. qRT-PCR analysis showed that they both express all 5 Notch ligands at detectable levels and no significant differences were observed between the monotypic LECs and cocultured LECs* (Supplemental Figure 7A). Of all the ligands, JAG1 and JAG2 showed the highest mRNA expression levels in LECs. To test which ligand on the LEC membrane mediates activation of Notch3 in the melanoma cells, we cultured different melanoma cell lines on cell culture dishes coated with immobilized chimeric proteins consisting of recombinant Fc fragments fused with the Notch ligands to mimic the pulling force of the ligand on the Notch receptor. After 2 days, cells were harvested and their mRNA levels of *NOTCH3* as well as Notch3 downstream targets *HEY1* and *HES1* were measured (Figure 6A). DLL4-Fc induced the expression of *NOTCH3* mRNA in the metastatic melanoma cell lines WM852 and WM165, while DLL1 induced *NOTCH3* only in WM165. Of the known Notch downstream targets, *HEY1* was upregulated in WM852 and WM165 by DLL4-Fc and to some extent by DLL1-Fc, albeit not significantly. We did not observe any clear changes in the expression level of *HES1*. The other tested Notch ligands did not induce any significant changes in *NOTCH3* or the downstream targets.

We also assessed the activation of Notch3 by immunoblotting. Culturing of the melanoma cell lines on the DLL4-Fc–coated dishes induced an increase in the cleaved, active NICD3, especially in the WM852 and WM165 cells and to a lesser extent in WM793 (Figure 6B and Supplemental Figure 7, B and C; see complete unedited blots in the supplemental material). NICD3 induction by DLL1-Fc coating was also seen in WM852 and WM165, but it was less pronounced compared with the DLL4-Fc–mediated induction. Interestingly, the full-length Notch3 receptor level was also increased by DLL1-Fc and DLL4-Fc treatments, which together with the observed increase in the mRNA levels (Figure 6A) suggest that Notch3 is induced through an autoregulatory loop in the melanoma cells.

Our previous report demonstrated that the LEC-induced Notch3 is crucial for the increased melanoma invasion (11). We next addressed the ability of DLL4-Fc to increase melanoma invasion by embedding the ligand-activated melanoma cells in a cross-linked 3D fibrin matrix for 4 days. According to our earlier results, DLL4-Fc could significantly increase the invasive growth of WM852 and WM165 melanoma cells into the surrounding fibrin (shown as invasive index in Figure 6C and Supplemental Figure 7D).

Lastly, we measured the *WNT5B* expression levels in the cells cultured on the chimeric Fc fragments–Notch ligands by qRT-PCR. DLL4-Fc coating induced an approximately 4-fold increase in *WNT5B* expression in WM852 and WM165 (Figure 6D). In WM165 cells, DLL1 and JAG2 also induced an approximately 2-fold increase in the *WNT5B* level, although it did not reach significance (Supplemental Figure 7E). In line with the earlier results, no significant changes in *WNT5B* expression were observed upon culturing the WM793 cells on top of the 5 different Notch ligands–Fc fragments (Figure 6D and Supplemental Figure 7E).

These results show that DLL4 on the LEC surface is a potent inducer of the Notch3 receptor on melanoma cells upon coculture, leading to increased invasion and upregulation of *WNT5B* in the metastatic melanoma cell lines.

## Discussion

**Figure 7:**
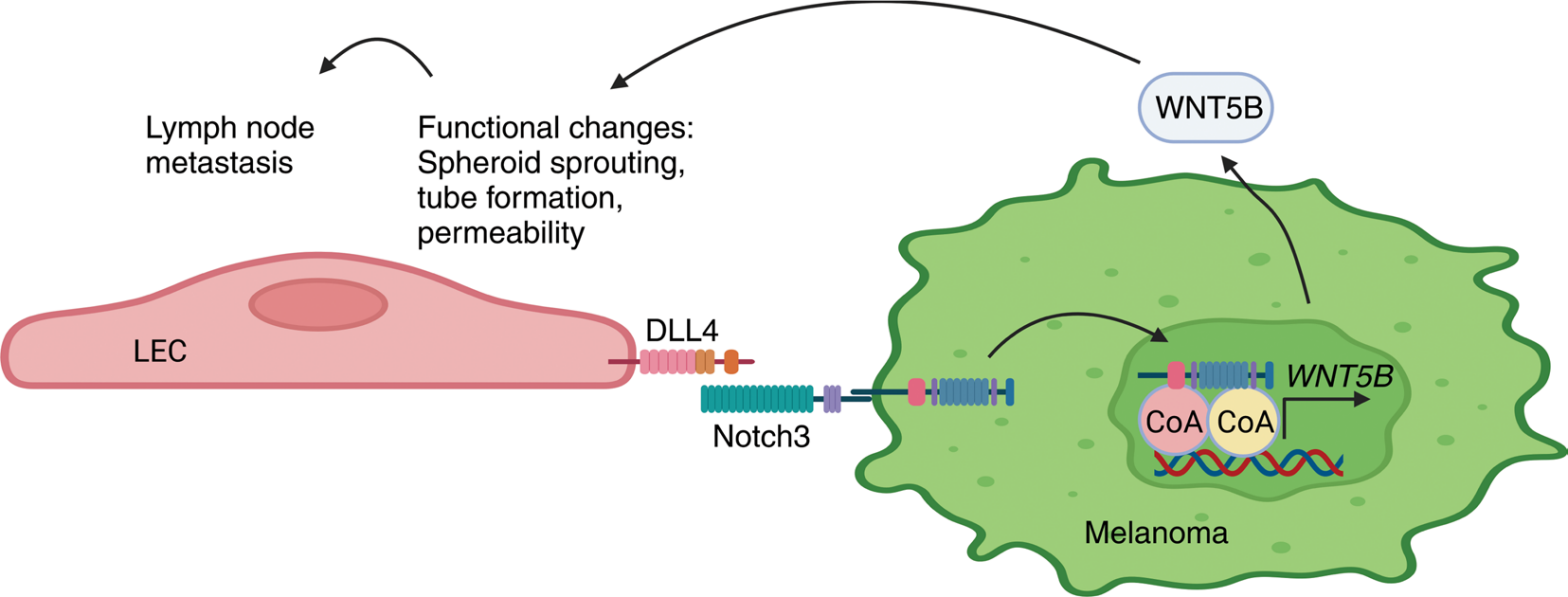
Model of the bidirectional melanoma cell–LEC crosstalk. Schematic model of the bidirectional melanoma cell crosstalk with LECs and the role of Notch3 in the LEC functional changes through induction of WNT5B.

Lymphatic endothelium has recently gained attention as an active, cancer-promoting element with functions beyond simply acting as a passive route for cancer cell dissemination. Here, we report that the DLL4/Notch3/WNT5B signaling axis induced upon melanoma cell and LEC interaction promotes melanoma metastasis via its effects on LEC phenotype and function. Specifically, we show that DLL4, expressed on the LEC surface, activates Notch3 in melanoma cells, which in turn triggers *WNT5B* transcription and protein expression in melanoma cells. The melanoma-derived WNT5B then contributes to the functional changes in LECs in a paracrine manner (Figure 7).

Following the melanoma coculture, we found upregulation of genes associated with inflammatory responses in the LECs*. These transcriptional changes are likely to contribute to the disruption of lymphatic junctions, thus leading to increased permeability of LEC monolayers and facilitating cancer dissemination. LECs can act both as the target of inflammatory signals and represent a remarkable source of tumor-promoting cytokines. As an example, it has been shown that tumor-exposed LECs significantly increase their IL-6 production, and thereby promote tumor cell invasion and proliferation (27). Our scRNA-seq data are in line with a previous study in mice where they found that pathways regulating immunomodulatory responses were upregulated in tumor-draining lymph node LECs compared with control naive lymph node LECs (28). Our scRNA-seq data revealed upregulation of genes involved in vascular developmental processes such as endothelin-1 and endoglin (29). Endothelin-1 produced by ECs has been shown to promote melanoma cell migration and vessel-like channel formation, and, interestingly, LECs cultured in the media containing endothelin-1 also show enhanced cell migration (30). Endoglin expression increases in ECs during vascular damage (31) and inflammation, and it facilitates the infiltration of inflammatory cells into the endothelium (32, 33). All these molecular changes along with additional gene expression changes found in our scRNA-seq data likely contribute to the tumor-promoting, phenotypic changes in LECs.

We identified WNT5B as a key component of the bidirectional, reciprocal melanoma cell–LEC crosstalk. WNT5B upregulation was mediated by Notch3 activation induced in melanoma cells by direct interaction with LECs upon coculture. Moreover, our results demonstrate that the melanoma-derived WNT5B participates in inducing the functional changes in LECs in a paracrine manner. The mouse ear pinna xenograft assay indicated that WNT5B facilitates the LEC-primed melanoma cell escape from the primary injection site and translocation to the proximate draining lymph nodes. Although WNT5B clearly contributes to melanoma cell escape from the initial injection site, other factors are also likely to contribute to this process; as an example, we found that the LEC-primed melanoma cells appear to grow as a more dispersed cell colony in a WNT5B-independent manner when compared with the monotypic cells. The dispersion is expected to result in more opportunities for melanoma cells to communicate with the mouse lymphatic vasculature in a WNT5B-dependent manner and thereby to promote lymphatic dissemination.

Our data also demonstrated that metastatic samples from a patient cohort with particularly aggressive melanoma showed a significantly higher correlation for Notch3 and WNT5B coexpression when compared with the primary tumors. This was further supported by mRNA analysis of a 442-patient melanoma data set obtained from TCGA, altogether supporting the importance of the Notch3/WNT5B axis in melanoma aggressiveness.

WNT5B, a member of the WNT family of proteins, has been described as an inducer of the noncanonical, β-catenin–independent WNT signaling pathways and is often considered an antagonist of canonical WNT signaling. It has been reported to regulate cell migration, proliferation, and differentiation during development (34). WNT5B is upregulated in triple-negative breast cancer patient samples and correlates with a worse prognosis (15). It is also associated with lung adenocarcinoma where its expression correlates with lymph node metastasis and poor survival (35). To our knowledge, the role of WNT5B in melanoma metastasis has not been previously characterized. Interestingly, in oral squamous cell carcinoma, WNT5B has been shown to be upregulated in tumor cells when compared with normal tissue (17). In addition, WNT5B was shown to induce partial endothelial-mesenchymal transition in LECs by increasing α-SMA expression and reducing VE-cadherin expression. Moreover, WNT5B depletion was shown to reduce lymph node metastases of oral squamous cell carcinoma cells, suggesting that WNT5B could be more broadly utilized by cancers to facilitate lymph node metastases (17). In melanoma mouse models it has been shown that metastasized melanoma cells in lymph nodes can invade the lymph node–associated blood vessels and disseminate directly to distant organs (36, 37). It is therefore possible that WNT5B is one of the key players contributing to the first metastatic steps in melanoma progression, and our results imply that melanoma cells require a direct interaction with LECs in the tumors for the initiation of the metastatic cascade.

We further found that Notch ligand DLL4, expressed in LECs, is a potent inducer among the tested ligands of Notch3 in melanoma cells. Notch3 is a central protein in changing melanoma cell behavior; its effects include induction of increased cell migration (38), stem-like cell characteristics (39), and invasion and metastasis (11). We and others have previously demonstrated that endothelial cells induce Notch3 expression in melanoma cells upon interaction (11, 38, 39). Here we show that Notch3 is not only contributing to the more aggressive melanoma cell characteristics but can also elicit functions that modify the melanoma tumor microenvironment, and thereby promote cancer progression. Accumulating evidence supports the contribution of Notch3 to the aggressiveness of melanoma. Therefore, it represents an appealing target for aggressive melanoma therapies. However, until now the clinical trials using Notch pathway inhibitors have not been successful, mainly due to the unspecific nature of currently available Notch inhibitors, which often lead to severe gastrointestinal side effects. Therefore, a deeper knowledge of the upstream and downstream effects of Notch3 signaling in melanoma may provide better cues for targetable molecular candidates. For instance, several phase I trials have been launched where Porcupine inhibitors were used to abolish WNT secretion from cancer cells, aiming at targeting cancer cell proliferation and cancer stem cells (40). These inhibitors might represent viable therapeutic modalities in targeting the tumor microenvironment as well.

Our study and other increasing evidence demonstrate that instead of simply acting as a route for metastatic and inflammatory cell transport, the lymphatic system is an active player shaping cancer cell behavior, and in turn the cancer cells can actively manipulate the lymphatic system to further promote tumorigenesis. Therefore, the bidirectional crosstalk and interaction between the tumor and lymphatic system can potentially provide viable molecular targets and opportunities to target cancer cell invasion and metastasis through combinatorial therapies.

## Methods

### Cell lines.

Primary human dermal LECs were obtained from Lonza and cultured in endothelial growth medium (EBM-2, Lonza) supplemented with growth factors included in the supplement bullet kit (except for the VEGF supplement) and 5% fetal calf serum (FBS; full media referred to as EGM-2). EGM-2 was also used to grow LEC–melanoma cell cocultures.

WM852, WM165, and WM793 melanoma cell lines (gifts from Kaisa Lehti, Norwegian University of Science and Technology, Trondheim, Norway) were cultured in Dulbecco’s modified Eagle medium (DMEM, Lonza) supplemented with 10% FBS and 1% penicillin/streptomycin. The cells were transduced with dual eGFP-luc (pMX-Rg) or GFP reporters (pLENTI6eGFP, Invitrogen) as described in Tatti et al. (41). All cultures were grown in standard conditions (37°C, 5% CO_2_) and regularly tested negative for mycoplasma using Eurofins Mycoplasmacheck service (https://eurofinsgenomics.eu/en/genotyping-gene-expression/applied-genomics-services/mycoplasmacheck/). The identity of the melanoma cell lines was authenticated by the Institute for Molecular Medicine, FIMM (University of Helsinki, Finland) cell line authentication service (https://www.helsinki.fi/en/infrastructures/genome-analysis/infrastructures/fimm-genomics/cellline-authentication).

### RNA interference and cell culture treatments.

Cells were cultured to approximately 60% confluence on 24- or 6-well plates and transfected with siRNAs at final concentrations of 10–25 nM using Lipofectamine RNAiMAX (Invitrogen) according to the manufacturer’s instructions. Cells were cultured in the presence of siRNA for 24 hours before using in subsequent assays. The following siRNAs were used: nontargeting control (Ambion, MA4390843; Dharmacon, D-001810-10-05), Notch3 (Ambion, 4392420; Dharmacon, L-011093-00-0005), WNT5B (Dharmacon, L-009761-00-0005), FZD6 (L005505-00-0005, Dharmacon), and FZD8 (L-003962-00-0005, Dharmacon).

Where indicated, γ-secretase inhibitor DAPT (Sigma-Aldrich) at 10 μM concentration and recombinant WNT5B protein (7347, R&D Systems) at 1000 ng/mL were applied.

### Transient transfection.

WM852 cells were seeded on 10-cm cell culture dishes 1 day prior transfection to reach 80%–90% confluence the next day. Cells were then transfected with 8.8 μg NICD3-pCLE (Addgene) using Fugene HD (Promega) according to the manufacturers’ instructions. Next day, the cells were used for subsequent analysis.

### Notch ligand coating.

Twenty-four– or 12-well plates were coated with the Fc domain, as a control, or Notch ligand fused with the Fc domain at a concentration of 10 μg/mL for 6 hours at room temperature (DLL1-Fc, 10184-DL-050 Biotechne; DLL3-Fc, DL3-H5255 ACRO Biosystems; DLL4-Fc, 158- 10171-H02H-100 Sino Biological; JAG1-Fc, 158-11648-H02H Sino Biological, and JAG2-Fc, 1726-JG-050 R&D Systems). Cells (0.4 × 10^5^ to 0.75 × 10^5^) were cultured on coated plates for 48 hours and then harvested and used for subsequent assays.

### Melanoma cell–LEC cocultures and cell sorting.

For the coculture of LECs with melanoma cells, cells were seeded on fibronectin-coated (Sigma-Aldrich) cell culture dishes in a 1:2 or 3:4 melanoma cell/LEC ratio in EGM-2 media. In the first experiments, LEC and melanoma cells were separated using magnetic nanoparticles. For this separation process, melanoma cells were first loaded with dextran-coated nanoparticles at 1 mg/mL (fluid-MAG-DX, Chemicell) for 24 hours. Dextran-loaded melanoma cells were cultured with LECs for 48 hours, after which the cells were sorted using a MidiMacs separator and LS columns (Miltenyi Biotec). Due to the cessation of the production of these magnetic nanoparticles, we had to change the separation to be carried out by FACS (Sony, SH800Z). The sorted cells were used for the subsequent functional or molecular assays.

### 3D fibrin assays.

The fibrin assays were adapted from a previously described angiogenesis assay (42), described in detail in Alve et al. (43). To study the melanoma cell invasion in 3D fibrin, 5000 melanoma cells were embedded in cross-linked fibrin (Calbiochem) and cultured for 72 hours. In the LEC spheroid assay, 5000 LECs were first seeded on a U-bottom 96-well plate precoated with 1% agarose (Invitrogen) overnight to let the LECs form spheroids under nonadherent conditions. The next day, the spheroids were harvested and embedded in a fibrin matrix. Four days later, fibrin droplets containing either the LEC spheroids or melanoma cells were fixed with 4% paraformaldehyde (PFA) and stained as indicated for subsequent analysis. The droplets were imaged using either a Zeiss LSM 780 confocal microscope or Nikon Eclipse TS2 phase contrast microscope and the images were analyzed with ImageJ software (NIH).

### Tube formation assay.

LECs (1 × 10^4^) were seeded on 96-well plates precoated with 50 μL Cultrex (3433, R&D Systems) and incubated 24 hours in the cell incubator. Phase contrast images of the cells, 4 fields per well, were obtained using the Nikon Eclipse TS2 phase contrast microscope and the tubular structures were quantified with ImageJ software.

### Electrical cell-substrate impedance assay.

LECs (2 × 10^4^) were seeded onto 10 mM L-cysteine– and 5 μg/mL fibronectin–coated 96-well plates (96W10idf, Ibidi) and the resistance of the cell monolayers was recorded at 4000 Hz by an ECIS Z Theta instrument connected to a computer running ECIS software v1.4.8 (Ibidi) over 4 days. The media were changed once during that time. The readout indicates the changes in resistance over time.

### RNA isolation and qRT-PCR.

A NucleoSpin II kit (Macherey-Nagel) was used to isolate RNA according to the manufacturer’s instructions. RNA (200–1000 ng) was reverse transcribed with the TaqMan reverse transcription kit (N8080234, Applied Biosystems). The transcript levels of *NOTCH3* were measured using a QuantiTect primer assay (QT00003374, Qiagen), and the sequences of the other targets are provided in Supplemental Table 4. The reactions were performed using SYBR Green PCR mix (Applied Biosystems).

### Western blot.

Western blot analysis was performed as previously described (11). Cells were lysed in RIPA buffer supplemented with protease and phosphatase inhibitors (Thermo Fisher Scientific). The protein amount of the lysates was measured with Bio-Rad protein assay dye reagent concentrate (Bio-Rad). Equal amounts of protein were loaded in 4%–15% SDS-PAGE gels (Bio-Rad) and the gels were run at 55 mA for 50 minutes. Proteins were transferred onto nitrocellulose membranes (Bio-Rad) using a Bio-Rad Trans-Blot Turbo and the membranes were blocked using 5% nonfat dry milk in Tris-buffered saline with 0.1% Tween 20 (TBST). The probing was done either for 1 hour at room temperature or overnight at 4°C using rabbit anti-Notch3 (M-143, Santa Cruz Biotechnology) or mouse anti–β-actin (A1978, Sigma-Aldrich) antibody. The primary antibody incubation was followed by incubation in HRP-conjugated secondary antibody for 1 hour at room temperature (goat anti–mouse IgG or goat anti–rabbit IgG, 7074 or 7076, Cell Signaling Technology). Bands were detected by chemiluminescence using ECL solution (WesternBright Sirius, Advansta) and visualized by Chemi-Doc (Bio-Rad).

### Immunofluorescent staining.

2D cell cultures were labeled as previously described (11) with antibodies against WNT5B (ab94914, Abcam), β-catenin (610153, BD Biosciences), ZO-1 (402200, Invitrogen), and VE-cadherin (555661, BD Biosciences). Secondary antibodies conjugated with Alexa Fluor 488, Alexa Fluor 594, or Alexa Fluor 647 (A21202, A21207, or A21447, Invitrogen) were used to visualize the labeled cells and the nuclei were counterstained with Hoechst 33342 (Fluka). The fluorescence images were acquired using a Zeiss LSM 780 confocal microscope and the sum of the pixel intensity values was quantified using CellProfiler (Broad Institute; https://cellprofiler.org/) and normalized to the number of cells.

### ChIP-PCR.

WM852 cells were transfected with the expression plasmid NICD3-pCLE (11) and 48 hours later, the chromatin was processed by the SimpleChIP kit according to the manufacturer’s instructions (Cell Signaling Technology). The chromatin was precipitated using antibodies against Notch3 (M-134, Santa Cruz Biotechnology or 8G5, Cell Signaling Technology) or normal goat or rat IgG antibody (sc-2028 or sc-2026, Santa Cruz Biotechnology). DNA was purified using the PCR Purification kit (Macherey-Nagel) according to the manufacturer’s instructions.

After DNA purification, the *WNT5B* promoter regions were amplified and analyzed by qPCR (Supplemental Table 4).

### Mouse ear pinna xenograft assay.

Female NOD.Cg-*Prkdc^scid^ Il2rg^tm1Wjl^*/SzJ mice (20 to 24 weeks old) were either purchased from Scanbur or obtained as a gift from Timo Otonkoski (University of Helsinki, Helsinki, Finland). Mice were housed in a temperature-controlled and pathogen-free facility with ad libitum access to food and water.

Melanoma cells (5 × 10^5^) were mixed in 1:1 PBS/Matrigel and injected intradermally into the ear pinna of the mice. Tumors were allowed to grow for 7, 8, or 14 days, after which the mice were sacrificed. The collected ears were split into ventral and dorsal halves and fixed in 4% PFA for 25 minutes at room temperature. Tumors were imaged with a Zeiss LSM 780 confocal microscope. The total areas covered by GFP-expressing melanoma cells were quantified by ImageJ.

Superficial cervical and inguinal lymph nodes were harvested, and tissue DNA was isolated using the NucleoSpin Tissue kit (Macherey-Nagel). The presence of human melanoma cells in the harvested mouse tissues was detected by quantitative PCR for the human Alu sequences, while the mouse actin sequence was used as control (Supplemental Table 4). For the FACS analysis, lymph nodes were dissociated to single-cell preparations using the protocol described in Broggi et al. (44). Isolated cells were then suspended in PBS and 30,000 cells were freshly analyzed using an Accuri C6 Plus analyzer (BD Biosciences). The lymph nodes from mice without tumor cell injection were used as a negative control for GFP gating.

### Clinical patient samples and immunohistochemistry.

Paraffin-embedded melanoma sections were provided by the Helsinki Biobank. All patients provided written informed consent to the Helsinki Biobank.

Tissue sections were deparaffinized and rehydrated. The EnVision FLEX+ kit (Dako, Agilent) was used to stain the tissues. Epitope retrieval was performed using the EnVision FLEX Target Retrieval solution, high pH, for 15 minutes at 95°C. Endogenous peroxidase blocking was performed according to the kit manufacturer. Tissue blocking was performed using the normal horse serum blocking solution (S2000, Vector Laboratories) diluted 1:20 in antibody diluent (S2022, Dako).

Sections were stained with either anti-Notch3 (1:800; HPA044392, Sigma-Aldrich) or anti-WNT5B antibodies (1:600; ab94914, Abcam) diluted in the Dako antibody diluent for 16 hours at 4°C. Secondary antibody incubation was performed according to the instructions of the kit. Chromogenic reactions were performed using the Romulin AEC Chromogen kit (RAEC810, Biocare Medical) and hematoxylin staining using Mayer’s Hematoxylin (S3309, Dako). Tissues were dehydrated and mounted on coverslips using Eukitt Quick-hardening mounting medium (03989, Sigma-Aldrich). The slides were imaged with a Pannoramic 250 Flash II (3DHISTEC Ltd.).

### TCGA data analysis.

The RSEM-quantitated mRNA-seq data along with the associated clinical data from TCGA Melanoma PanCancer Atlas data set were used to perform Kaplan-Meier survival association analysis with the log-rank method and Pearson’s correlation analysis. SPSS v29.0.1.0 (IBM) was used to perform the analyses and generate the graphs.

### scRNA-seq.

LECs* sorted from WM852 melanoma cocultures (sample 2) were mixed with control cells consisting of LECs and WM852 melanoma cells, both from monotypic cultures, in a ratio of 10:1 (sample 1), from 3 replicates. The monotypic LECs and WM852 cells were mixed to identify the potential, residual melanoma cells remaining in sample 2 after separation using magnetic beads in the cocultured LECs and WM852. The cells were processed according to the 10× Genomics guidelines (https://www.10xgenomics.com/support/single-cell-geneexpression/documentation/steps/sample-prep/single-cell-protocols-cell-preparation-guide). The Single Cell 3′ Reagent kit v2 (10× Genomics) was used to label each cell and transcript with a unique molecular identifier (UMI) and to generate the cDNA library. Sequencing was performed using an Illumina NovaSeq 6000 sequencer. Data postprocessing and quality control were performed with 10× Genomics Cell Ranger (v2.1.1) software.

The filtered count matrices were preprocessed and clustered using the Seurat v4.3.0 tool in an RStudio v2023.03.0 environment (R v4.2.3; https://satijalab.org/seurat/). Briefly, the filtered count matrices were imported using the Read10X function and transformed into Seurat objects, filtering out cells with less than 200 features and features encountered in less than 3 cells. Percentages of mitochondrial (pt.mito) and ribosomal RNAs (pt.rRNA) were calculated and cells were further filtered according to the following: (a) control data: 2000 < nFeature_RNA < 6000, pt.mito < 10, 3 < pt.rRNA < 40; and (b) cocultured sample data: 1000 < nFeature_RNA < 4500, pt.mito < 10, 3 < pt.rRNA < 45. The final Seurat objects comprised 53.9% (2792 cells, control) and 30.5% (4970, coculture) of the raw cell counts. Next, the data were normalized on a logarithmic scale of 10,000, and the 2000 most variable features were retrieved using the FindVariableFeatures function, applying the method of variance stabilizing transformation.

Data anchors were calculated between the control and coculture samples using FindIntegrationAnchors and 50 dimensions, and thereafter were integrated using the IntegrateData function. The data were scaled, regressing for RNA counts (nCount_RNA), pt.mito, and pt.rRNA, and principal component analysis was performed using the integrated assay. Clusters were calculated using 30 dimensions for the FindNeighbors function and a resolution of 0.6 for FindClusters, which resulted in 9 final clusters denoted from cluster 0 to 8. Using the RNA assay, cluster markers were calculated based on the control data set using the FindMarkers function with a minimum detection rate of 0.25.

The integrated Seurat object was subsetted into control and coculture samples, and the individual objects were further subsampled to have equal cell numbers according to the smallest number of cells encountered in the control sample (2700 cells). Then, the subsampled objects were merged back together using the merge function. Differentially expressed genes (DEGs) were calculated by comparing the subsampled coculture and control data using the FindMarkers function, with a log(fold change) threshold set to 0.25. Upregulated genes in the coculture versus control samples with an adjusted *P* value below 0.05 were used for gene ontology (GO) analysis using the ShinyGO v0.77 web platform (http://bioinformatics.sdstate.edu/go/). Here, default parameters were used to calculate the top 20 GO biological processes related to the gene lists.

### Statistics.

Data were analyzed with Prism 9 (GraphPad) The graphs show the mean across the biological replicates and the error bars indicate SD. The number of experimental groups is indicated in figure legends. One-way ANOVA or 2-tailed *t* test was performed to assess the statistical significance of the differences between samples. *P* values lower than 0.05 were considered significant.

The ECIS data were analyzed by calculating the area under the curve (AUC) using Prism 9. One-way ANOVA or 2-tailed *t* test was performed to assess the significance of the differences between AUCs.

For the patient sample immunohistochemical analysis, corresponding bulk cancer tissue areas of both Notch3 and WNT5B samples were assessed for the expression of the respective proteins using a 10× magnification field from the 3DHISTECH-digitized samples, covering an area of approximately 3.75 mm^2^ per sample. Samples with observable staining were scored as positive if a meaningful area (>0.2 mm^2^) within the sample was stained. The correlation percentage was calculated for both the primary sample and metastasis sample data sets, including both positive and negative correlations between Notch3 and WNT5B. A paired-sample *t* test was used to assess statistical significance of the difference in colocalization numbers between primary and metastasis sample sets, utilizing samples for which both Notch3 and WNT5B were successfully scored from both the primary and metastasis samples.

### Study approval.

Paraffin-embedded melanoma sections were provided by the Helsinki Biobank. All patients provided their written informed consent to the Helsinki Biobank. The study protocol and the use of the material was approved by The Ethics Committee of the Hospital District of Southwest Finland (HUS/206/2022). All the animal experiments were approved by the Committee for Animal Experiments of the District of Southern Finland (license ESAVI/10548/2019).

### Data availability.

Values for all data points in graphs are reported in the Supporting Data Values file. The scRNA-seq data have been deposited in the NCBI Gene Expression Omnibus (GEO) and are accessible through GEO Series accession number GSE247542 (https://www.ncbi.nlm.nih.gov/geo/query/acc.cgi?acc=GSE247542).

## Author contributions

SA, SG, and PMO conceptualized the study. SA, SH, and EM performed experiments. SJ and AAM designed and collected the patient cohort and curated the patient samples and data as dermatopathologists. SA, JJ, and EM performed formal analysis. SA, MHL, and SK performed analysis of the scRNA-seq data. OC, PS, and PMO provided resources. AP, SK, and KV provided guidance for experiments. SG and PMO supervised the study. PMO provided funding. SA and PMO wrote the original draft of the manuscript, and SK and KV contributed to editing the text.

## Supplementary Material

Supplemental data

Supplemental table 1

Supplemental table 2

Supplemental table 3

Supplemental table 4

Supporting data values

## Version 1 11/16/2023

In-Press Preview

## Version 2 01/09/2024

Electronic publication
