# Supplementary material for: DLL4/Notch3/WNT5B axis mediates bidirectional prometastatic crosstalk between melanoma and lymphatic endothelial cells
Source: JCI Insight. 2024 Jan 9;9(1):e171821. doi: 10.1172/jci.insight.171821 (PMC10906450; doi:10.1172/jci.insight.171821)
Supplement: Supplemental table 3 [file jciinsight-9-171821-s100.pdf]

Supplemental data table III: Clinical parameters of a melanoma cohort consisting of sentinel lymph node biopsy negative, recurrent cases

	N (%)	MSS HR (95 % CI)	RFS HR (95 % CI)
RFT (Years)			
Mean	2.35		
Median	2.08		
Range	0.42 - 5.92		
Follow-up (years)			
Mean	4.33		
Median	3.75		
Range	1.17-8.25		
Outcome			
Alive	7 (12.7)		
Died of melanoma	42 (76.4)		
Died of other disease	6 (10.9)		
Site of first metastasis			
Skin/sotf tissue	22 (48.9)		
Lymph node	15 (33.3)		
Distant organ			
Total	8 (17.8)		
Brain	4 (8.9)		
Lung	3 (6.7)		
Liver	1 (2.2)		
Age (years)			
Mean	61.82	0.0369 (1.002-1.05)	
Median	61		
Range	31-88		
Gender			
Female	14 (38.9)	NS	NS
Male	22 (61.1)		
Location			
Trunk	19 (34.6)	NS	NS
Lower Limb	16 (29.1)		
Upper Limb	7 (12.7)		
Head and neck	13 (23.6)		
Breslow thickness			
< 2mm	11 (20.0)	NS	NS
2.00 - < 4.00 mm	25 (45.5)		
4.00+ mm	19 (34.5)		
Clark			
III	16 (29.1)	NS	NS
IV	32 (58.2)		
V	7 (12.7)		
Ulceration			
Absent	26 (47.3)	0.046 (1.011 - 3.828)	NS
Present	20 (36.4)		
N/A	9 (16.4)		
Dermal mitoses			
<1/mm2	16 (29.1)	NS	NS
≥1/mm2	27 (49.1)		
N/A	12 (21.8)		
BRAF V600E			
Negative	29 (52.7)	NS	NS
Positive	20 (36.3)		
NA	6 (10.9)		

Abbreviations:
MSS HR = Melanoma specific survival hazard ratio p-value (assessed via Cox Regression analysis),
RFS HR = Recurrence free survival hazard ratio p-value (assessed via Cox regression analysis),
95 % CI = 95 % confidence interval for statistically significant variables in the Cox regression analyses,
NS = statistically not significant (limit for statistical significance: p-value < 0.05),
RFT = Recurrence free time,
Clark = Clark depth of primary tumor,
BRAFV600E = presence of constitutively activating BRAF V600E missense mutation assessed at the clinic via immunohistochemical staining.
